# Spencer’s Objectives for Microscopes

**Published:** 1852-08

**Authors:** 


*Spencer's Objectives for Microscopes.*—The pre-eminent success of Chas. A. Spencer, residing in Canastota, Madison county, New York, in manufacturing objectives for microscopes, deserves a notice in this place. It is now fairly conceded that Spencer, though an American, has considerably excelled the best English and European opticians in this most difficult department of practical optics. The American Association for the Advancement of Science have given him this award, and English microscopists have borne testimony to the same effect.

The preceding observations were made with a glass of exquisite workmanship, one of Spencer's latest and best productions. I gave him an order for the finest objective of high power which he could make, expressly without limit as to price. He sent me the instrument [the essential parts of which the smallest thimble would contain] in May, 1852, writing me at the same time that it was the best he had ever made, and charging me for it, what I consider a most moderate sum, $120; for its defining power is so great and so wonderfully accurate, that a sum of money greater than I choose to name, would not deprive me of its possession. It is rated by Spencer as one-sixteenth of an inch focus, though the available working focal distance is probably less than 1-200th of an inch, requiring the very thinnest of Chance's thin glass, for covering objects to be seen. Its angle of aperture is full 174°! a figure at least forty units beyond what the best European opticians have, until quite lately, considered practicable. Upon this, as well as upon the general perfection of workmanship, its great excellence depends. That most beautiful test object, the Grammatophora subtilissima, of Bailey, is, by this glass, readily and clearly resolved into black beaded lines.—*Dr. J. L. Riddell, in New Orleans Med. and Surg. Jour.*

